# Metabolic interactions underlying phyllosphere microbiota assembly

**DOI:** 10.1093/ismejo/wraf155

**Published:** 2025-07-28

**Authors:** Wenzhuo Feng, Fengquan Liu

**Affiliations:** State Key Laboratory of Green Pesticides, Department of Plant Pathology, College of Agriculture, Guizhou University, Guiyang, Guizhou 550025, P. R. China; State Key Laboratory of Green Pesticides, Department of Plant Pathology, College of Agriculture, Guizhou University, Guiyang, Guizhou 550025, P. R. China

**Keywords:** phyllosphere microbiota, metabolic interactions, NOI, GEMs, cross-feeding

## Introduction

The phyllosphere is the Earth’s largest microbial habitat and is critical for plant health and biogeochemical cycles [1]. The assembly of microbial communities depends on metabolic networks, abiotic stresses (ultraviolet, desiccation, and oxidative stress), and plant antimicrobials. Additionally, resource competition and cross-feeding interactions are key structural drivers [1]. However, the exact mechanisms underlying these processes remain unclear. A fundamental unanswered question is: how can predictive theoretical models be constructed to reliably simulate interspecies metabolic interactions?

The classical niche differentiation theory provides a foundational understanding of microbial ecological interactions. As a key example, obligate methylotrophs (*Methylobacterium* spp.) and metabolically versatile heterotrophs (*Sphingomonas* spp.) exhibit clear spatial partitioning driven by divergent substrate utilization strategies. However, traditional assessment tools such as the Niche Overlap Index (NOI) are limited. While useful for predicting competitive exclusion in simplified systems (among *Pseudomonas* spp., *Escherichia coli,* and *Corynebacterium glutamicum* under defined carbon conditions), they struggle with complex networks involving multi-substrate synergy and cross-feeding via nutrient exchange and byproduct recycling [2]. Overcoming these limitations requires novel frameworks that integrate genomic functional potential into ecological theory, which is now prioritized through computational biology.

Genome-scale metabolic models (GEMs) have transformed microbial interaction studies by providing an advanced computational platform that facilitates system-level analysis through the systematic integration of species-specific gene (protein) associations, metabolite transport kinetics, and environmental constraint parameters [3]. Unlike purely empirical or statistical approaches such as Bayesian and neural networks, GEMs use a mechanistic foundation rooted in biochemical reaction stoichiometry and thermodynamics, enabling the quantitative prediction of metabolic fluxes via techniques such as flux balance analysis and dynamic modeling [3]. GEMs have been used to successfully resolve metabolic interactions in diverse ecosystems. For example, rhizosphere models incorporating root exudate dynamics predicted plant-microbe co-dependence in carbon-nitrogen cycling [4], whereas gut microbiome GEMs revealed host diet-dependent vitamin cross-feeding networks [5].

Although agent-based models capture spatial heterogeneity and cellular individuality (biofilm structural dynamics), GEMs provide further strengths by resolving molecular-level constraints on metabolic exchanges. Similarly, ecological network models focus on the topological properties of species interactions (connectivity patterns), whereas GEMs quantitatively predict metabolite fluxes and species-specific metabolic strategies. A prominent example is the delineation of the core metabolic differences between *Firmicutes* (superior acetate producers) and *Proteobacteria* (preferred monosaccharide consumers) [5]. The ongoing enhancement and multiscale integration of these network-based modeling approaches are constructing a solid theoretical foundation for deciphering the fundamental rules that regulate microbial metabolic interactions.

Recently, Schäfer *et al.* published a detailed study that improves our understanding of phyllospheric metabolic interactions [6]. Using the *Arabidopsis thaliana* Leaf Surface Bacterial Resource (At-LSPHERE) containing 224 bacterial isolates, 45 distinct carbon substrate utilization profiles were systematically characterized. Their analysis revealed a deep phylogenetic conservation of metabolic functions, identifying glucose as the most widely utilized carbon source, whereas degradation of aromatic compounds showed a restricted distribution. Metabolic consistency was preserved at the genus level, with Beta- and Gamma-proteobacteria displaying high metabolic flexibility, which differed markedly from the narrow substrate ranges observed in Actinobacteria and Bacteroidetes. Methylotrophs display striking specialization and are spatially associated with methanol-rich microhabitats, strongly supporting niche differentiation via volatile organic compound metabolism [1]. However, the evolutionary foundation of this metabolic conservation remains unclear. Current explanations are limited to vertical gene inheritance or niche specialization. Nonetheless, fundamental questions remain: Does phylogenetic conservation primarily result from vertical transmission of core metabolic pathways? To what extent do environmental factors influence the conserved metabolic patterns across taxonomic groups? Horizontal gene transfers or convergent evolution may account for the observed exceptions. Resolving these questions requires combining comparative genomics, functional validation, and ecological modeling to clarify the evolutionary origins and ecological drivers of these conserved traits. Although bacterial communities dominate current phyllosphere models, emerging evidence indicates that fungal-bacterial secondary metabolites exchange and phage-mediated nutrient recycling fundamentally reshape metabolic landscapes [7, 8].

Schafer *et al.* (2023) developed the At-LSPHERE-GEMs framework by systematically integrating carbon substrate utilization profiles, achieving 89% predictive accuracy (balanced accuracy: 0.98 ± 0.07) [6]. These findings empirically demonstrate that including ecologically relevant parameters significantly enhances the predictive capability of GEMs. This improvement primarily stems from the rigorous manual refinement of metabolic pathways, which reduces false-positive predictions caused by genome annotation errors [3]. While manual refinement increases model reliability, it risks introducing researcher bias, highlighting the need for standardized quality assurance protocols such as MEMOSys [9]. Machine learning methods are now proving essential for improving the accuracy and automation of GEM predictions [9]. Future studies should focus on creating machine-learning-enhanced and dynamically optimized GEMs tailored specifically for phyllosphere microbial communities.

**Figure 1:**
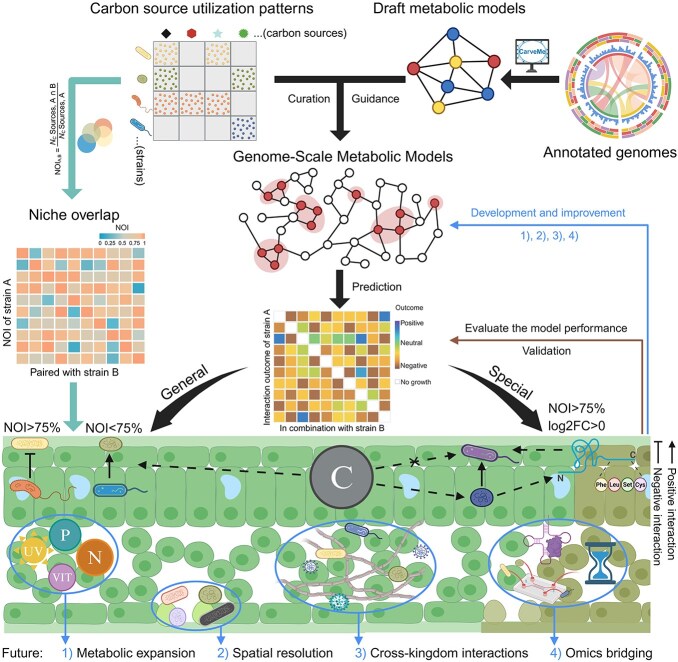
Schematic framework for predicting phyllosphere microbial carbon utilization and metabolic interactions, integrating niche differentiation mechanisms. This schematic integrates four key components: (upper left) carbon source utilization patterns analyzed through the NOI, with a threshold of NOI = 75% distinguishing competition-dominated from resource-partitioning dynamics; (Center) GEM construction, developed through annotation-based genome analysis and manual curation with carbon utilization data, capable of predicting general scenarios (NOI-aligned) and special cases (NOI < 75%, log2FC > 0 or cross-feeding, depicted by dashed arrow networks). The “C” circle represents carbon source substances. The two connectors denote negative and positive interactions, respectively. Dashed arrows indicate metabolic capabilities, and the right-angle polyline arrow indicates that plant experiments can verify and evaluate the performance of the models; (bottom) future research priorities outline key steps to enhance the predictive accuracy and applicability of the model. The figure was created with the software BioRender (BioRender.com).

To verify the computational model predictions, Schafer et al. constructed synthetic microbial consortia (SynCom7 and SynCom3) comprising strains with different metabolic capacities for plant colonization studies [6]. The results showed that 93% of the antagonistic interactions occurred under high niche overlap conditions (NOI > 0.75), where metabolically limited strains underwent systematic suppression. This validates the niche theory principle wherein resource competition drives competitive exclusion when ecological niches overlap significantly. Conversely, metabolically versatile strains displayed significant growth advantages (log2FC > 0) in the low-NOI consortia, showing strong concordance with model predictions (Fig. 1). The substantial decrease in positive interactions observed in batch cultures highlights the inherent constraint of laboratory conditions for mimicking continuous nutrient fluxes and the microscale spatial heterogeneity of natural phyllospheric environments. This result demonstrates the superiority of the computational framework in precisely simulating in situ ecological interactions [6]. These observations extend the niche theory by showing how cross-feeding-mediated complementarity can mitigate competition under resource scarcity, explaining coexistence despite overlap.

A detailed examination of microbial resource competition strategies revealed that ~63.2% of pairwise interactions exhibited antagonistic relationships, whereas only 25.5% showed strong positive outcomes. Moreover, 94% of the interactions involved growth suppression of at least one partner, demonstrating the dominance of competitive exclusion over facultative mutualism in phyllosphere communities [6]. Furthermore, context-dependent facilitative behaviors emerged in high-NOI consortia through metabolic flux redistribution; during carbohydrate scarcity, metabolically versatile strains increased amino acid and organic acid assimilation to maintain biomass production and mitigate competitive stress [6]. However, metabolic versatility alone failed to ensure positive outcomes in low-adaptability strains such as *Exiguobacterium*. Limited carbon utilization was circumvented when *Acinetobacter* secretions stimulated antimicrobial synthesis from leucine and glutamate via cross-feeding [10]. This conditional cooperative advantage aligns with the niche differentiation theory, though further validation is required via metabolomic tracing of Acinetobacter-derived amino acids and targeted genetic perturbations (BCAT disruption and NRPS knockout). While current models incorporate spatial and phylogenetic dimensions, critical limitations persist in temporal dynamics (diel nutrient oscillations), incomplete substrate coverage (omitting key metabolites like dihydroxyacetone and γ-aminobutyrate), and exclusion of uncultivated functional taxa such as ammonia-oxidizing archaea and auxotrophic candidate phyla radiation bacteria, which limits the assessment of nitrogen cycling dynamics and dependent cross-feeding interactions.

The GEMs refined by Schäfer *et al.* established a strong framework that bridges the ecological theory and mechanistic modeling. Subsequent research should prioritize: (i) Integrating supplementary physiological variables such as abiotic stress responses, nitrogen/phosphorus cycling, and vitamin dependencies to extend niche theory beyond carbon metabolism; (ii) Decoding stomatal nutrient gradients via spatially resolved modeling to quantify microhabitat effects on niche partitioning; (iii) Broadening investigations to encompass interkingdom relationships between bacteria, fungi, and viruses to uncover fundamental microbial governance of community homeostasis; (iv) Integrating multiscale metabolomics combining in situ profiling with temporal mapping, functional genomics for dependency validation and metagenomic reconstruction, and microfluidics-informed spatial modeling to bridge molecular mechanisms with ecosystem-level predictions in microbiome engineering (Fig. 1). Collectively, this integrated methodological framework will enable comprehensive elucidation of metabolic interaction networks governing microbiome community assembly.

## Data Availability

Data sharing is not applicable to this article as no datasets were generated for the current study.
